# The role of selenium metabolism and selenoproteins in cartilage homeostasis and arthropathies

**DOI:** 10.1038/s12276-020-0408-y

**Published:** 2020-08-13

**Authors:** Donghyun Kang, Jeeyeon Lee, Cuiyan Wu, Xiong Guo, Byeong Jae Lee, Jang-Soo Chun, Jin-Hong Kim

**Affiliations:** 1 Center for RNA Research, Institute for Basic Science, Seoul, 08826 South Korea; 2 Department of Biological Sciences, College of Natural Sciences, Seoul National University, Seoul, 08826 South Korea; 3 School of Public Health, Xi’an Jiaotong University, Xi’an, 710061 China; 4 Interdisciplinary Program in Bioinformatics, Seoul National University, Seoul, 08826 South Korea; 5 National Creative Research Initiatives Center for Osteoarthritis Pathogenesis and School of Life Sciences, Gwangju Institute of Science and Technology, Gwangju, 61005 South Korea

**Keywords:** Osteoarthritis, Mechanisms of disease

## Abstract

As an essential nutrient and trace element, selenium is required for living organisms and its beneficial roles in human health have been well recognized. The role of selenium is mainly played through selenoproteins synthesized by the selenium metabolic system. Selenoproteins have a wide range of cellular functions including regulation of selenium transport, thyroid hormones, immunity, and redox homeostasis. Selenium deficiency contributes to various diseases, such as cardiovascular disease, cancer, liver disease, and arthropathy—Kashin–Beck disease (KBD) and osteoarthritis (OA). A skeletal developmental disorder, KBD has been reported in low-selenium areas of China, North Korea, and the Siberian region of Russia, and can be alleviated by selenium supplementation. OA, the most common form of arthritis, is a degenerative disease caused by an imbalance in matrix metabolism and is characterized by cartilage destruction. Oxidative stress serves as a major cause of the initiation of OA pathogenesis. Selenium deficiency and dysregulation of selenoproteins are associated with impairments to redox homeostasis in cartilage. We review the recently explored roles of selenium metabolism and selenoproteins in cartilage with an emphasis on two arthropathies, KBD and OA. Moreover, we discuss the potential of therapeutic strategies targeting the biological functions of selenium and selenoproteins for OA treatment.

## Joint health: the rare micronutrient selenium may be crucial

Selenium, a micronutrient found in brazil nuts, shiitake mushrooms, and most meats, may aid in treating joint diseases, including the most common form of arthritis, osteoarthritis (OA). In addition to thyroid hormone metabolism and immunity, selenium is important in antioxidant defense. Oxidative damage can destroy cartilage and harm joints, and selenium deficiency is implicated in several joint diseases. Jin-Hong Kim at Seoul National University in South Korea and co-workers reviewed selenium metabolism, focusing on OA and and Kashin–Beck disease, a skeletal development disorder prevalent in selenium-deficient areas of northeast Asia. They report that selenium-containing proteins protect cells against oxidative damage and that selenium is crucial to cartilage production. Further investigation of selenium metabolism may point the way to new treatments for OA and other joint diseases.

## Introduction

Selenium (Se) is an essential trace element in humans^1,2^. Selenium is generally taken up from the diet through food or other forms of external supplementation. Dietary selenium is obtained in the form of selenomethionine (SeMet), selenocysteine (Sec), selenite, and selenate. Significant health benefits have been attributed to selenium metabolic systems that play major physiological roles in thyroid hormone metabolism, immunity, and antioxidant defense^2,3^. Selenium is required for the production of thyroid hormone-metabolizing enzymes and selenium supplementation is thought to improve the function of thyrocytes and immune cells^4^. Selenium supplementation demonstrated immunostimulant effects, such as enhanced proliferation of activated T cells, activation of natural killer cells, and tumor cytotoxicity mediated by cytotoxic lymphocytes^5,6^. In contrast, selenium deficiency is associated with the occurrence, virulence, and disease progression of viral infections^7^.

Selenium inadequacy can lead to various types of diseases, most notably cardiovascular disease^8–12^, cancer^13–15^, hepatopathy^16,17^, and arthropathy. Cardiovascular diseases are associated with systemic selenium level, with a higher risk at <55 or >145 μg/L selenium concentration in the blood^10^. A type of endemic cardiomyopathy, Keshan disease is linked to selenium deficiency^8,11^. Keshan disease occurs in low-selenium areas in China and is prevented by sodium selenite supplementation^12^. Low-selenium status is correlated with a significantly increased risk of cancer incidence and mortality^13–15^. Epidemiological studies have provided evidence on the cancer-preventing effects of selenium^18–20^. Selenium deficiency is also characterized by elevated levels of oxidative stress markers in the liver^21^, which significantly contribute to liver injury^17^. The oxidative stress caused by selenium deficiency further plays a detrimental role in joint development. Selenium deficiency is the main cause of endemic Kashin–Beck disease (KBD), which is mainly reported in low-selenium areas of China, North Korea, and the Siberian region of Russia. Moreover, there is a growing body of evidence suggesting that the pathogenesis of osteoarthritis (OA), the most common form of arthritis, may be associated with selenium deficiency by resulting in oxidative stress^22–28^. However, it is noteworthy that excessive selenium intake can also cause selenosis^29,30^, which accompanies adverse symptoms including fatigue, diarrhea, nausea, increased heart rate, necrosis in liver and kidney, and neurological damage. Chronic selenosis eventually compromises immune and reproductive systems in humans.

OA is characterized by progressive loss of cartilage extracellular matrix (ECM) and pathological changes in other joint tissues such as subchondral bone sclerosis, osteophyte formation, and synovial inflammation^31^. Cartilage destruction is considered a hallmark of OA and is a result of increased production of catabolic effectors^32–35^ and reduced matrix biosynthesis by chondrocytes^36^. OA is associated with multiple etiologies involving systemic factors such as age^37^ as well as local factors such as mechanical stress^38^ driven by weight-bearing and joint instability. Both OA-causing factors have been found to cause oxidative stress in chondrocytes. Oxidative stress results from the abnormal production of reactive oxygen species (ROS) and the loss of cellular antioxidant capacity. Many preclinical and clinical studies have indicated the accumulation of oxidative burden in chondrocytes undergoing osteoarthritic changes^39,40^. Emerging evidence suggests that oxidative stress is mechanistically linked to the initiation of osteoarthritic changes in chondrocytes through the acquisition of senescent phenotypes^36^. Therefore, restoring redox homeostasis can serve as a rational therapeutic strategy to alleviate OA progression. Here, we review the role of selenium metabolism in cartilage and bone and the significance of maintaining its homeostasis in the context of joint diseases such as KBD and OA.

## Overview of the selenium metabolic system

### The selenium metabolic system and the biosynthesis of selenoproteins

**Fig. 1:**
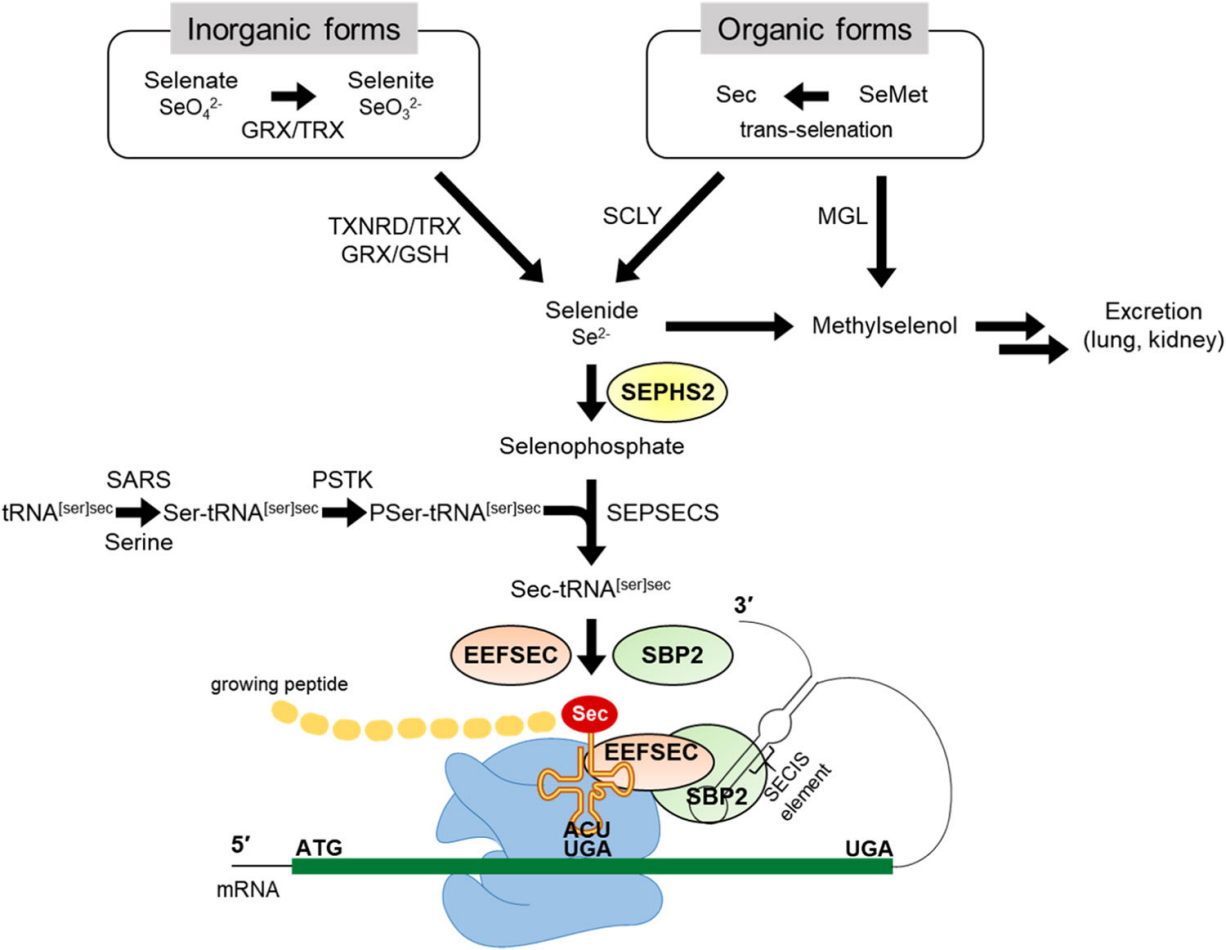
Selenium metabolic system in mammals. Selenium is absorbed from the diet, undergoes several conversion steps, and is incorporated into polypeptide chains, completing selenoprotein synthesis. Dietary sources of selenium uptake exist in inorganic form such as selenate and selenite and organic form such as Sec and SeMet. Inorganic forms are reduced by TXNRD/TRX or GRX/GSH systems and organic forms are cleaved by SCLY, forming selenide. Selenophosphate is synthesized from selenide by SEPHS2, and the subsequent reaction with PSer-tRNA^[Ser]Sec^ mediated by SEPSECS yields Sec-tRNA^[Ser]Sec^. Sec-tRNA^[Ser]Sec^ is transferred to the A-site of ribosome mediated by SBP2, which binds to SECIS located in the 3′UTR of a selenoprotein mRNA. Finally, the UGA codon is recognized as the Sec integration codon. Abbreviations: SeMet, selenomethionine; Sec, selenocysteine; GRX, glutathione reductase; TRX, thioredoxin; TXNRD, thioredoxin reductase; GSH, glutathione; MGL, methionine gamma-lyase; SCLY, selenocysteine lyase; SEPHS2, selenophosphate synthetase 2; SARS, seryl-tRNA synthetase; PSTK, phosphoseryl(Sep)-tRNA kinase; SEPSECS, Sep-tRNA:Sec-tRNA synthase; EEFSEC, Sec-specific eukaryotic elongation factor; SBP2, SECIS binding protein 2.

Selenium metabolism is a systemic process that includes the absorption, transportation, transformation, and excretion of selenium (Fig. 1). Selenium is obtained in organic forms—SeMet and Sec—and inorganic forms—selenite and selenate—from diet. Selenium is taken up by the liver that synthesizes and exports SELENOP which eventually circulates through the bloodstream. SELENOP, with multiple Sec residues^41^, transports selenium to other tissues and organs^42^ and the transported selenium is converted to selenophosphate by intracellular selenium metabolic pathways. Selenium is excreted through exhalation and urine in the form of small-molecule metabolites formed by sequential methylation^43,44^.

Selenium plays biological roles predominantly in the form of selenoproteins synthesized by the selenium metabolic system. Ingested inorganic selenium is first reduced to hydrogen selenide (H_2_Se) via glutathione (GSH) and thioredoxin (TXN) systems. Selenide is further converted to Sec amino acids for incorporation into specific sites of selenoproteins such as the catalytic sites of a selenoenzyme. Mechanistically, selenophosphate synthetase 2 (SEPHS2) catalyzes the production of selenophosphate through the reduction of hydrogen selenide. The subsequent reaction with phosphoseryl-tRNA (PSer-tRNA^[Ser]Sec^) yields Sec-tRNA^[Ser]Sec^. Sec amino acids are incorporated into polypeptide chains through the machinery utilizing the UGA codon. Selenocysteine insertion sequence binding protein 2 (SBP2) binds to selenocysteine insertion sequence (SECIS) element which is located in the 3′-untranslated region (3′UTR) of selenoprotein mRNA, and mediates the transfer of Sec-tRNA^[Ser]Sec^ to the A-site of ribosome which recognizes the UGA codon as the Sec integration codon. Collectively, the selenoprotein translation machinery consists of SECIS element, SBP2, Sec-specific eukaryotic elongation factor (EEFSEC), and aminoacylated Sec-tRNA^[Ser]Sec^, thereby enabling UGA to be recognized as a Sec codon and utilized for translation into the growing polypeptide.

### Selenoproteins

Selenoprotein is defined as a protein containing Sec amino acid residue. The biological functions of selenium are mostly exerted through selenoprotein domains that contain Sec residues. Twenty-five selenoprotein genes have been identified in the human genome^45^. In mice, a total of 24 selenoproteins have been characterized^46^ and targeted deletion of some of these selenoproteins demonstrated their essential roles in developmental processes and in disease pathogenesis. Selenoproteins can be classified into subfamilies based on their cellular functions such as those implicated in antioxidation (GPX1, GPX2, GPX3, GPX4), redox regulation (TXNRD1, TXNRD2, TXNRD3, MSRB1, SELENOH, SELENOM, SELENOW), thyroid hormone metabolism (DIO1, DIO2, DIO3), selenium transport and storage (SELENOP), selenophosphate synthesis (SEPHS2), calcium metabolism (SELENOK, SELENOT), myogenesis (SELENON), protein folding (SELENOF, SELENOI, SELENOS), and protein AMPylation (SELENOO)^47,48^. The functions of other selenoproteins such as GPX6 and SELENOV still remain unclear. Glutathione peroxidases (GPXs) such as GPX1 (cytosolic GPX), GPX2 (gastrointestinal GPX), and GPX4 (phospholipid hydroperoxide GPX) catalyze the decomposition of a great variety of peroxides, thus protecting cells against oxidative damage^49,50^. Thioredoxin reductases (TXNRDs) employ NADPH as an electron donor to revert oxidized TXN to a reduced dithiol, the oxidation status of which is critically implicated in regulating various cell behaviors including proliferation and apoptosis^51^. The physiological significance of TXNRDs is further supported by the embryonic lethality of *Txnrd1* or *Txnrd2* knockout mice^52,53^. Deiodinases (DIOs) regulate thyroid hormone metabolism by catalyzing the conversion of thyroid hormones from precursor thyroxine (T_4_) to biologically active triiodothyronine (T_3_) or inactive reverse T_3_ (rT_3_)^54^. The expression levels of several selenoproteins are influenced by the extent of selenium uptake. For example, selenium-deficient animals and human cell lines exhibit reduced transcription of selenoproteins such as *GPX1*, *DIO*s, *SELENOI*, and *SELENOW*^55–57^. A subset of selenoproteins such as GPX1 and SELENOW is more sensitive to selenium supplementation or deficiency. The hierarchy of selenoprotein expression is more apparent when the intracellular level of selenium is limited^1^.

### Selenium-responsive genes

Selenium-responsive genes are the genes whose expression patterns are influenced by supplementation with selenium or selenium-containing compounds. Treatment of a cancer cell line with methylseleninic acid induced expression changes in 951 genes^58^. These responsive genes were closely associated with annotations related to cell cycle regulation, androgen-responsive genes, and phase II detoxification pathway. Selenium supplementation of macrophages diminished the expression of lipopolysaccharide (LPS)-induced pro-inflammatory genes such as cyclooxygenase-2 (COX-2) and tumor necrosis factor-α (TNF-α)^59^, suggesting that selenium has anti-inflammatory effects on the immune system. The CTD database (http://ctdbase.org/) reports the effect of environmental chemicals including selenium on gene expression profiles in various human tissues.

## The role of selenium and selenoproteins in cartilage development and KBD

### Selenium levels and its role in joint tissues

Joints are composed of various types of connective tissues including cartilage, bone, synovium, meniscus, and ligament. Among these tissues, cartilage is the main component that absorbs mechanical stress, cushioning bones from impacting each other during various weight-bearing activities. In the human knee joint, the selenium concentration in cartilage is approximately 80 μg/kg dry weight, whereas the selenium concentrations in ligament and meniscus are 270 and 307 μg/kg dry weight, respectively^60,61^. The requirement of adequate physiological selenium levels for maintaining cartilage homeostasis has been recognized. Selenium deficiency retards the growth and development of cartilage and bone^62–66^. Growth retardation was observed in rats after two generations of selenium deficiency^62^. Mice fed a diet deficient in selenium resulted in fibrocartilage formation at the articular surface, ultimately showing degeneration of articular cartilage^63^. Selenium deficiency induced the expression of the chondrocyte hypertrophy marker gene type X collagen (COLX) in articular cartilage^64^. The expression of parathyroid hormone-related protein (PTHrP), which controls chondrocyte maturation during endochondral ossification, was enhanced in both articular cartilage and hypertrophic growth plate following selenium deficiency. These changes were in line with the phenotypic changes observed in the cartilage of KBD patients^64^. However, it should be noted that growth retardation caused by selenium deficiency may also be associated with the deregulation of bone metabolism^65^. In a study by Cao et al., selenium deficiency severely compromised bone microarchitecture as a result of increased bone resorption^66^.

### Abnormalities in selenium metabolism and skeletal development diseases

Selenium deficiency is regarded as one of the initiating factors of KBD, which is an endemic osteoarthropathy caused by the premature closure of epiphyseal plate and the impaired skeletal development. Skeletal deformities in hands, fingers, knees, and elbows, and in severe cases, dwarfism and movement disorders are the symptoms of KBD^22^. The KBD area roughly coincides with low-selenium areas including a geological belt extending from northeast to southwest China, North Korea, and eastern Siberia^22^. A meta-analysis showed that selenium levels in the water, soil, cereal, and corn in KBD endemic regions were lower than they were in non-endemic regions, supporting the fact that the level of selenium in tissue is predominantly affected by dietary intake^23^. In line with this finding, selenium levels in the whole blood, serum, hair, and urine of KBD patients were markedly lower than those of healthy controls^24^.

Selenoprotein gene polymorphisms are associated with increased susceptibility to KBD. There were significant differences in the allelic frequency of *GPX1* Pro198Leu (rs1050450) between the KBD and control group^67^. In addition, the mRNA level of *GPX1* and enzyme activity of total GPX in blood were lower in the KBD group than they were in the control group^67^. Haplotypes of TCC, TTC, and TTT of rs1050450, rs3811699, and rs1800668 in *GPX1* gene also had a significant link to KBD^68^. A single-nucleotide polymorphism (SNP) in the promoter region of *SELENOS* rs28665122 (−105G > A) was related to the increased risk of KBD and upregulation of PI3K/Akt signaling in patients with KBD^69^. In this study, tert-butyl hydroperoxide (tBHP) treatment-induced chondrocyte apoptosis was mitigated by selenium supplementation via sodium selenite treatment, which suppressed the PI3K/Akt pathway. The minor A-allele of *SELENOF* rs5859 was associated with a significantly higher incidence of KBD^70^.

The animals fed a selenium-deficient diet recapitulated some of the pathological manifestations of KBD, strongly supporting the notion that selenium deficiency is critically associated with the development of this endemic arthropathy. Selenium deficiency impaired bone and cartilage growth with the exhibition of premature chondrocyte hypertrophy as evidenced by an increased expression of COLX, compatible with the phenotypes in KBD cartilage^64^. The low-selenium condition in combination with three mycotoxins, deoxynivalenol (DON), nivalenol (NIV), and T-2, yielded pro-catabolic changes and hypertrophic phenotype of chondrocytes, as evidenced by the loss of aggrecan and type II collagen (COLII) and the increase in COLX and matrix metalloproteinases (MMPs) expression, respectively^71^. In contrast, selenium supplementation partially alleviated these mycotoxin-induced damages in chondrocytes^71^. In rats, dietary selenium deficiency over two generations caused the onset of physiological selenium insufficiency^72^. In this condition, pathological changes in the epiphyseal plate were observed with the decreased expression of COLII and GPX1 in the chondrocytes, suggesting a possible association of reduced chondrocyte anabolism and antioxidant capacity with the epiphyseal plate lesions observed in KBD^72^. The relevance of impaired selenium metabolism to the onset of KBD was further validated using a mouse genetic deletion model. Targeted deletion of Sec-tRNA^[Ser]Sec^ (*Trsp*) gene in osteochondroprogenitor cells from embryonic stage caused the depletion of selenoproteins in skeletal systems, causing growth retardation, abnormalities in the epiphyseal growth plate, delayed endochondral ossification, and chondronecrosis, which recapitulated the major pathological features of KBD^73^.

As a prophylactic treatment, selenium supplementations were given to children living in a KBD area. The supplemented group showed elevated physiological selenium levels in their hair samples and exhibited a substantially lower prevalence of KBD^74^. A meta-analysis including five randomized controlled trials (RCTs) and ten prospective non-RCTs statistically demonstrated the benefits of selenium supplementation in preventing KBD in children^75^.

## Selenium metabolism and OA

### Physiological significance of oxidative stress in chondrocytes

OA is the most common form of arthritis and is primarily characterized by the loss of cartilage-specific ECM and other pathological changes in joints including subchondral bone sclerosis, osteophyte formation, and synovial inflammation^31^. Articular cartilage is composed of abundant proteoglycans in which sulfated glycosaminoglycan chains such as chondroitin sulfates are bound to a core protein such as aggrecan. Loss of cartilage matrix during OA progression is a combined result of increased catabolic process in cartilage and reduced anabolic activity of chondrocytes. The molecular-level understanding of OA pathogenesis has led to the identification of major catabolic enzymes, ADAMTS5^76^, MMP3^77^, and MMP13^78^, which mediate the degradation of cartilage matrix. Pro-inflammatory cytokines drive the expression of these catabolic factors in chondrocytes through the activation of transcription factors such as HIF-2α^32^ and NF-κB^79^. Abnormalities in various metabolic pathways such as glucose^80^ or amino acid metabolic system^81^ in chondrocytes have been implicated in activating catabolic cascades in osteoarthritic cartilage^82^. Moreover, increased cellular uptake of Zn^2+^ through the upregulation of zinc transporter ZIP8 activates metal-regulatory transcription factor-1 (MTF1), which in turn induces the expression of matrix-degrading enzymes in chondrocytes^33,83^. Regulation of catabolism by the cholesterol–CH25H–CYP7B1–RORα axis further showed the association of metabolic abnormalities with the catabolic process of OA^34^.

Meanwhile, the upstream regulatory mechanism eliciting an imbalance in OA matrix homeostasis needs further investigation. OA-causing factors such as age and mechanical stress lead to excessive oxidative stress in chondrocytes^37,38^. Consistently, clinical and preclinical OA studies indicated a cumulative oxidative burden in osteoarthritic chondrocytes^39,40^. Emerging evidence suggests that oxidative stress plays a significant role in OA development and the disease progression can be mitigated by counteracting oxidative stress^36,84–86^. In general, oxidative stress results from the abnormal production of ROS and the loss of cellular antioxidant capacity. Synovial fluid from patients with late-stage OA who were undergoing knee joint replacement had a lower level of oxidoreductases than that from healthy controls^87^. In part, the increase in oxidative stress is attributable to mitochondrial dysfunction in OA chondrocytes^88,89^. OA chondrocytes displayed reduced mitochondrial DNA content, mitochondrial dysfunction, and diminished expression of NRF2 which regulates the transcription of oxidoreductase genes^89^. Similarly, chondrocytes from aged individuals exhibited increased ROS burden and mitochondrial and genomic DNA damage^90–92^. Therefore, the proper maintenance of redox homeostasis can potentially serve as a rational therapeutic strategy to protect against OA progression.

### Potential roles of selenium metabolism in OA

The protective effect of selenium in OA has been explored in a large number of epidemiological and genetic studies (Table 1). The concentration of selenium in serum was significantly lower in OA patients than that of normal controls^25^. Similarly, the results from a population-based cohort study demonstrated the linkage between low-selenium levels in toenails with OA-associated pain and disease severity^26,27^. Several studies have indicated that cartilage matrix homeostasis is impaired in selenium deficiency. Low-selenium status diminished COLII expression level regulated by SOX9, which is known as a master regulator required for maintaining cartilage matrix homeostasis. In fact, SOX9 was destabilized by the downregulation of selenium-responsive PRMT5 that sustains SOX9 stability via methylation^93^. In another study, rats fed a selenium-deficient diet exhibited low sulfotransferase activity, which resulted in diminished contents of sulfated glycosaminoglycan essential for mechanical stress-absorbing property of cartilage matrix^28^. In contrast, selenium supplementation ameliorated the spontaneous degeneration of articular cartilage in STR/1 N mice by increasing the expression of GPXs^94^. In cultured chondrocytes, pretreatment with SeMet markedly inhibited nitric oxide (NO) and prostaglandin E2 (PGE2) production in response to pro-inflammatory cytokine IL-1β^95^. Expression of *SBP2*, a factor recognizing SECIS element, had a positive correlation with *GPX1* and *GPX4* expression and antioxidant capacity in chondrocytes^96^. Oxidation resistance mediated by SBP2 was diminished in response to IL-1β treatment in vitro and in damaged regions of cartilage in OA patients^96^. Downregulation of selenoprotein mRNAs including *GPX3*^97^, *GPX1*, and *GPX4*^96,98^, and *Selenop*^99^ was observed in human and mouse OA chondrocytes.

**Table 1:** List of selenoproteins associated with the pathogenesis of arthropathies, KBD and OA.

Gene	Function	Expression in OA	SNP	Ref.
GPX1	Antioxidant Reduction of hydrogen peroxide and organic peroxides	Downregulated	rs1050450 (KBD) rs3811699 (KBD) rs1800668 (KBD)	^67,68,96,98^
GPX3	Plasma antioxidant	Downregulated		^97^
GPX4	Detoxification of lipid hydroperoxides Metabolism of lipids	Downregulated		^96,98^
DIO2	Activation of hormones Deiodination of T_4_ to T_3_	Upregulated	rs225014 (OA) rs12885300 (OA)	^101–104^
DIO3	Inactivation of hormones Conversion of T_4_ to rT_3_		rs945006 (OA)	^105^
SELENOF	Protein folding		rs5859 (KBD)	^70^
SELENOP	Storage and transport of Se Antioxidant properties	Downregulated		^99^
SELENOS	Protein folding ER-associated protein degradation		rs28665122 (KBD)	^69^

Genetic factors such as SNPs in selenoproteins were identified to be risk factors for OA development. A GAG haplotype in *SELENOS* gene was significantly associated with increased levels of inflammatory factors in OA patient plasma^100^. SNPs in *DIO2*, which converts precursor thyroid hormone T_4_ to its active form T_3_, were also related to genetic susceptibility to OA development. Levels of *DIO2* mRNA and protein were markedly upregulated in OA cartilage^101^. A common *DIO2* haplotype composed of the minor C-allele of SNP rs225014 and the common C-allele of SNP rs12885300 was significantly associated with advanced hip OA, as indicated by a higher odds ratio^101–103^. Locus rs225014, which confers risk to OA, was associated with the differential methylation of CpG located in the upstream region of *DIO2* gene and was correlated with upregulated *DIO2* expression in OA^104^. Meanwhile, DIO3 depletes the resources that can be utilized for the production of active thyroid molecules by catalyzing the conversion of T_4_ and T_3_ into inactive metabolites. The minor G-allele of the *DIO3* variant rs945006 was associated with a protective effect against OA development^105^.

However, a few aspects regarding the relationship between selenium and OA remain controversial. First, several studies indicate that there are no differences in selenium levels between OA and normal tissues. The selenium concentrations in synovial fluid and plasma of 25 OA patients were not significantly different from those of 25 healthy controls^106^. Similarly, no significant difference in selenium concentration was noted between six dogs with post-traumatic OA and six control dogs^107^. Second, the beneficial effect of selenium supplementation in alleviating OA symptoms has been debated. The results from a controlled double-blind trial of 30 patients revealed that the supplementation of a formulation containing selenium with vitamins A, C, and E (Se-ACE) did not have any remarkable curative effect compared to a placebo^108^. In a study with an independent cohort, the prevalence of radiographic knee OA was not significantly associated with dietary selenium intake^109^.

Nonetheless, it is apparent that selenium deficiency, dysregulation of selenoproteins, and genetic variations in selenoprotein genes serve as potential risk factors for OA. The vital role of selenium metabolism in maintaining cartilage homeostasis is expected, considering its critical involvement in regulating cellular processes such as chondrogenic differentiation of progenitor cells, maintenance of redox homeostasis and DNA damage repair in chondrocytes, which are covered in the next section.

## Intracellular roles of selenium metabolism and selenoproteins in cartilage

### Chondrogenic differentiation programs of progenitor cells

Selenium exerts various beneficial effects to promote proliferation and differentiation of chondrogenic progenitor cells^110,111^. Selenium supplementation stimulated the proliferation of ATDC5 chondrogenic cells even under serum deprivation by inducing cyclin D1 expression^110^. Deficiency of SELENOO interfered with the chondrogenic differentiation of ATDC5 cells by suppressing the expression of chondrogenic genes SOX9, COLII, and aggrecan and decreasing the activity of alkaline phosphatase^112^. Knockdown of *Gpx1* reduced the chondrogenic differentiation of ATDC5 cells by modulating intracellular GSH/oxidized GSH (GSSG) ratio^113^. *Selenop* was differentially upregulated during the chondrogenic differentiation of micromass culture of mesenchymal cells isolated from mouse limb buds^114^. In line with the effects of selenium metabolism and selenoproteins in chondrogenic progenitor cells observed in vitro, deficient uptake of selenium severely affected chondrogenic differentiation of mesenchymal lineage cells and thus endochondral ossification in mice^64^. Osteochondroprogenitor-specific deletion of *Trsp* gene significantly impaired chondrogenic programs, causing abnormalities in bone and cartilage development in mice^73^.

### Antioxidant defense and redox homeostasis

The protective effects of selenium on cartilage are primarily attributed to the function of antioxidant defense^115–117^. The metabolism and survival of chondrogenic progenitors and chondrocytes are greatly compromised by ROS including free radicals, peroxides, and superoxide anions^118–120^. Recent studies strongly support the notion that mitochondrial dysfunction and oxidative stress are the main drivers of OA pathogenesis^37^. Although ROS play essential roles in the maintenance of basal cellular activities such as chondrocyte proliferation and matrix remodeling in cartilage, excessive oxidative stress causes detrimental events such as cellular senescence^36,121^, dedifferentiation^122^, and apoptosis^123^. ROS cause oxidative damage to various cellular components and disrupt the balance between ECM catabolism and anabolism^119^. ROS suppress mitochondrial oxidative phosphorylation and ATP production, which are essentially required to sustain cartilage matrix synthesis^124^. In addition, ROS induce matrix degeneration through the upregulation of matrix-degrading enzyme expression while this effect is abolished by antioxidant treatment^123,125^. The detrimental effects of ROS on cartilage homeostasis can be effectively alleviated by augmenting cellular antioxidant activity under stress conditions, and several attempts have been made to treat OA by targeting the regulators involved in oxidative stress production in cartilage^84–86^.

The protective role of selenium metabolism is thought to be exerted through the neutralization of ROS via antioxidant activities of selenoproteins including GPXs and TXNRDs. Bone marrow stromal cells cultured in medium supplemented with low selenite concentration exhibited ROS accumulation along with the reduced expression of GPXs, TXNRDs, and other selenium-independent oxidoreductase enzymes, resulting in micronuclei generation which is an indication of chromosome damage^126^. Both GPX1 expression and activity were substantially lower in mice fed a selenium-deficient diet than those in mice fed a normal diet, leading to decreased trabecular number, reduced femoral trabecular volume/total bone volume ratio, and trabecular separation^66^. The rats exposed to a selenium-deficient diet with T-2 toxin showed increased lipid peroxidation level and decreased antioxidant GPX activity in their serum and cartilage^127^. A selenium-deficient diet induced the expression of miR-138-5p, which in turn suppressed the expression of *SELENOM* that has antioxidant function, and caused mitochondrial dysfunction and apoptosis of chondrocytes^128^. Lead (Pb)-induced oxidative stress and toxicity reduced the expression of selenoprotein mRNAs, and the effect was mitigated by selenium supplementation^129^. In summary, the antioxidant properties of selenoproteins showed therapeutic potential by counteracting the accumulation of damage induced by oxidative stress in cartilage.

### DNA damage repair

It is well known that DNA damage pathways play substantial roles in the progression of arthropathies^119^. The expression of genes related to DNA damage was changed in the cartilage of KBD patients^130,131^. Chronic DNA damage induces the initiation of apoptosis or cellular senescence in chondrocytes^36,132,133^. Selenium has a potential to reduce DNA damage and increase DNA repair capacity^134^. In part, the beneficial effect of selenium on genomic stability is associated with the antioxidation effect of selenoproteins such as GPXs and TXNRDs, which remove ROS before they cause DNA damage^134^. Cancer cells supplemented with selenium (30 nM sodium selenite or 10 μM SeMet) showed elevated levels of GPX1 and TXNRD1 enzyme activity, effectively protecting against DNA strand breaks induced by ultraviolet A- or H_2_O_2_-induced oxidative stress^135^. SeMet reduced the extent of DNA damage and enhanced DNA repair capacity by inducing repair complex formation in DNA-damaged cells through UV radiation exposure^136^. SeMet treatment elevated the levels of p53 and REF1 proteins and induced their interaction with BRCA1^137^, resulting in the activation of DNA repair pathways. Cells treated with SeMet showed significantly enhanced DNA repair capacity under exposure to various DNA damaging agents such as UV radiation or cisplatin treatment^138^.

### Conclusion and perspectives: selenium metabolism-based therapeutic strategies for treating arthropathies

**Fig. 2:**
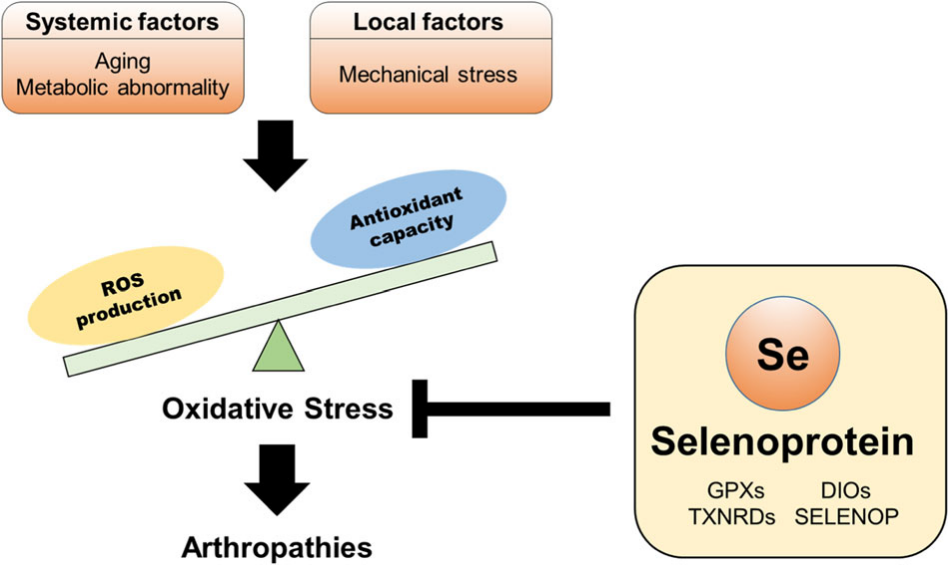
The protective function of selenoproteins against imbalanced redox homeostasis and the progression of arthropathies. Oxidative stress induced by systemic and local factors causes arthropathies, and selenoproteins play protective roles in the maintenance of redox homeostasis. OA, one of the most common forms of arthropathies, is related to multiple etiologies involving systemic factors and local factors such as aging, metabolic abnormality, and mechanical stress associated with overuse, injury, and misalignment. These OA-causing factors disrupt the balance between ROS production and antioxidation, thereby resulting in accumulation of oxidative stress. The dysregulation of redox homeostasis causes the disruption of cartilage homeostasis and leads to the development of arthropathies such as KBD and OA. Restoring redox homeostasis through the activation of selenium metabolism and supplementation with selenoproteins can be a rational therapeutic strategy to treat arthropathies.

This review discussed the roles of selenium metabolism in cartilage development and arthropathies such as KBD and OA, and highlighted its crucial functions in maintaining cartilage homeostasis. Considering the physiological role of selenoproteins in antioxidation and the detrimental effects of oxidative stress in chondrocytes, aberrant selenium metabolism is likely to disrupt cartilage homeostasis and cause arthropathic diseases via dysregulation of redox homeostasis (Fig. 2). Besides the protective role against oxidative stress, selenium appears to exert pro-anabolic effects to augment the regeneration capacity of cartilage. Selenium is essentially required to induce the proliferation and chondrogenic differentiation of mesenchymal stem cells. However, the mechanisms by which selenium metabolism regulates chondrogenic programs remain still unclear and require further investigation. Despite the overall beneficial effects of selenium in maintaining cartilage homeostasis, strategies to supplement selenium or selenoproteins should be considered with care to avoid adverse health effects such as selenosis. Strategies aimed at optimizing the benefits of selenium and selenoproteins should be considered for the therapeutic treatment and prevention of arthropathies.
